# Elevated Monoamine Oxidase-A in Anterior Cingulate of Post-Mortem Human Parkinson’s Disease: A Potential Surrogate Biomarker for Lewy Bodies?

**DOI:** 10.3390/cells11244000

**Published:** 2022-12-10

**Authors:** Jogeshwar Mukherjee, Reisha M. Ladwa, Christopher Liang, Amina U. Syed

**Affiliations:** Preclinical Imaging, Department of Radiological Sciences, University of California-Irvine, Irvine, CA 92697, USA

**Keywords:** monoamine oxidase-A, Parkinson’s disease, Lewy bodies, ubiquitin, [^18^F]FAZIN3, fluoroethyl harmol, PET, fluorine-18, autoradiography, QuPath

## Abstract

Lewy bodies (LB) play a neuropathological role in Parkinson’s disease (PD). Our goal was to evaluate LB using anti-ubiquitin immunohistochemistry (UIHC) and find correlations with monoamine oxidase-A (MAO-A) using imaging agent, [^18^F]FAZIN3. Human post-mortem anterior cingulate (AC) and corpus callosum (CC) from control subjects (CN), *n* = 6; age 81–90 LB = 0 and PD, *n* = 6, age 77–89, LB = III–IV were sectioned (10 μm slices). Brain slices were immunostained with anti-ubiquitin for LB (UIHC) and analyzed using QuPath for percent anti-ubiquitin per unit area (μm^2^). Adjacent brain slices were incubated with [^18^F]FAZIN3 and cortical layers I–III, IV–VI and CC (white matter) regions were quantified for the binding of [^18^F]FAZIN3. UIHC was correlated with [^18^F]FAZIN3 binding. All PD brains were positively UIHC stained and confirmed presence of LB. Outer cortical layers (I–III) of PD AC had 21% UIHC while inner layers (IV–VI) had >75% UIHC. In the CN brains LB were absent (<1% UIHC). Increased [^18^F]FAZIN3 binding to MAO-A in AC was observed in all PD subjects. [^18^F]FAZIN3 ratio in PD was AC/CC = 3.57 while in CN subjects it was AC/CC = 2.24. Increases in UIHC μm^2^ correlated with [^18^F]FAZIN3 binding to MAO-A in DLU/mm^2^. Increased [^18^F]FAZIN3 binding to MAO-A in PD is a potential novel “hot spot” PET imaging approach.

## 1. Introduction

Parkinson’s disease (PD) is the second most common neurodegenerative disease after AD and is the most common movement disorder. Currently, about 2% of the population over the age of 60 is affected. PD is a proteinopathy; it is characterized by the accumulation and aggregation of misfolded α-synuclein [1]. Neuropathological hallmarks are intracellular inclusions containing α-synuclein LB and Lewy neurites and the loss of dopaminergic neurons in the substantia nigra of the midbrain and in other brain regions as well. PET imaging of PD has been extensively studied using [^18^F]FDG and dopaminergic radiotracers [2]. Loss of dopaminergic neurons is not the only neuropathological alteration in PD, as microglial activation and an increase in astroglia are also known. An increase in microglial activation was shown in minipigs to be an early response to the accumulation of α-synuclein in the absence of dopamine neuron degradation [3]. Thus, PD is now recognized to have an inflammatory component [4].

Lewy bodies are always found in the substantia nigra and other specific brain regions in PD. They are mainly composed of structurally altered neurofilaments and occur wherever there is excessive loss of neurons. The age-specific prevalence of LB ranges from 3–8% to 12–8% between the sixth and ninth decades [5]. LB disease can be presymptomatic in cases of PD and the importance of age and time has been confirmed in the evolution of the disease. LB formation involves interactions between α-synuclein aggregates and membranous organelles, including mitochondria, and is one of the major drivers of neurodegeneration through disruption of cellular functions, inducing mitochondria damage and deficits and synaptic dysfunctions [6]. Mitochondrial dysfunction and damage is thus strongly associated with LB pathology [7].

Upregulation of MAO-A expression due to α-synuclein aggregates has been reported [8]. MAO-A inhibitors are used as a treatment for affective disorders [9]. More selective, reversible inhibitors for MAO-A were developed in order to avoid the adverse effects of irreversible inhibition, namely the cheese effect [10]. Harmine, acting on MAO-A, has been proven to have anti-depressant effects theorized to come from its ability to restore BDNF levels and increase hippocampal neurogenesis [11]. Moclobemide, a MAO-A inhibitor, is an antidepressant that has no tyramine effect and is comparable to tricyclic amines and serotonin reuptake inhibitors and has been studied in elderly patients with cognitive decline and depression [12]. Thus, if MAO-A is indeed upregulated in PD, suitable MAO-A inhibitors may be considered early in the treatment of PD. Recent work shows MAO-A metabolizes dopamine more than MAO-B does and thus MAO-A has a greater regulatory influence on dopamine [13,14].

Reversible MAO inhibitors have been used for brain disorders ranging from PD to affective disorders [10]. There are fewer drugs for MAO-A compared to MAO-B. Irreversible PET imaging agents for MAO-A such as [^11^C]clorgyline [15] have been extensively studied in substance abuse and we have made the irreversible radiotracer [^18^F]fluoroclorgyline for studies of antidepressant, fluoxetine [16,17]. Reversible PET imaging radiotracers for MAO-A are necessary for reliable quantitation and there is now ongoing effort to develop such MAO-A PET imaging agents [18]. Reversible MAO-A PET radiotracer [^11^C]Harmine has been used to study depression [19] and [^18^F]FEH [20] has only been used in animal imaging. Quantitative human PET studies using [^11^C]harmine have been carried out [21]. To the best of our knowledge, PET imaging of MAO-A in PD has not been reported.

The sporadic nature of PD development can make studying specific pathologies challenging [7,22]. Aggregation of misfolded α-synuclein in intracellular inclusions and LB are some of the hallmarks of human PD [23]. Formation of LB includes misfolded, aggregated α-synuclein, membranous organelles, mitochondria damage and other cellular components leading to synaptic dysfunction and cell death [24]. Electron micrographs of LB have shown the presence of mitochondria surrounding the LB [6].

In anterior cingulate (AC), LB accumulation may be used to predict cognitive deficits in PD [25]. These LB contain mitochondria which may be normal or dysphoric in the neuron of PD subjects and are likely to contain MAO-A in the outer layers of the mitochondria [26]. Previous PET imaging studies have shown high levels of MAO-A in the anterior cingulate of normal human volunteers [21]. Since AC contains both LB and MAO-A, we envisaged that AC would be a good brain region to study any alterations or associations of MAO-A and LB. These findings could potentially provide support for MAO-A as a surrogate marker for LB. If successful, MAO-A PET imaging could be developed as a diagnostic imaging tool for the presence of LB in PD. Thus, in this study we evaluated LB in human postmortem AC in PD and control (CN) subjects using anti-ubiquitin immunohistochemistry (UIHC) [27] and correlated them to MAO-A using the selective PET imaging agent, [^18^F]FAZIN3.

## 2. Materials and Methods

### 2.1. General Methods

Clorgyline and (*R*)-deprenyl were purchased from Research Biochemicals (Sigma Aldrich, St Loius, MO, USA). All other chemicals were obtained commercially from Sigma Aldrich, St. Louis, MO, USA. All solvents used were provided by Fisher Scientific. Fluorine-18 autoradiographic studies were carried out by exposing tissue samples on storage phosphor screens (Multisensitive, Medium MS phosphor screens, Perkin Elmer, Waltham, MA, USA ). The apposed phosphor screens were read and analyzed by OptiQuant acquisition and analysis program of the Cyclone Storage Phosphor System (Packard Instruments Co., Boston, MA, USA).

### 2.2. Human Brain Specimens

Human postmortem brain tissue samples were obtained from Banner Sun Health Research Institute (BHRI), Sun City, AZ brain tissue repository for in vitro experiments. Age and gender matched PD brain and cognitively normal (CN) brain tissue samples were selected for end-stage pathology [1,28]. Human postmortem brain slices (10 μm) were obtained from chunks of frozen tissue on a Leica 1850 cryotome, cooled to −20 °C and stored at −80 °C. The brain slices containing anterior cingulate and corpus callosum (controls (CN), *n* = 6; age 81–90 LB = 0 and PD, *n* = 6, age 77–89, LB = III–IV) were used. Semi-quantitative scores of none, sparse, moderate and frequent were converted to numerical values 0–3 for each brain region. Numerical values 0–3 for each region were summed to provide total LB. All PD brains had a primary diagnosis of PD with some clinical history of dementia. Adjacent slices were used for immunostaining with anti-ubiquitin and anti-α-synuclein. All postmortem human brain studies were approved by the Institutional Biosafety Committee of University of California, Irvine.

### 2.3. Anti-Ubiquitin and α-Synuclein Immunohistochemistry

Immunostaining of all brain sections were carried out by University of California-Irvine, Pathology services using Ventana BenchMark Ultra protocols. Neighboring slices were immunostained for Ubiquitin (Cell Marque catalog no. 318A-18, Rocklin, CA, USA) and α-synuclein (EMD Millipore Corporation, lot No. 2985418, Burlington, MA, USA). All IHC stained slides were scanned using the Ventana Roche instrumentation and analyzed using QuPath. Adjacent slices were also immunostained for Aβ plaques and total Tau. All IHC stained slides were scanned using the Ventana Roche instrumentation and analyzed using QuPath [29].

### 2.4. [^18^F]FAZIN3 and [^18^F]FEH

The radiosynthesis of [^18^F]FAZIN3 was carried out in house. Fluorine-18 in H_2_^18^O from PETNET was used to react with the precursor, AZIN3 Tosylate (1–2 mg in 0.5 mL of anhydrous acetonitrile) for 15 to 30 min at 96 °C. Product was purified in a reverse-phase HPLC C_18_ Econosil column 250 mm × 10 mm (Alltech Assoc. Inc., Deerfield, IL) with 60% acetonitrile: 40% water containing 0.1% triethylamine with a flow rate of 2.5 mL/min. Retention time of [^18^F]FAZIN3 was found to be 10.5 min. Batches of ~370 MBq [^18^F]FAZIN3 were produced in specific activity of >74 TBq/mmol, and was stable for in vitro studies. Solvents from the HPLC purified fraction were removed and [^18^F]FAZIN3 was taken in 5 mL of 0.9% sterile saline and passed through a 0.22 micron Millipore filter into a sterile dose vial.

Using a two-step procedure, [^18^F]fluoroethyl harmol ([^18^F]FEH) was produced. *N*-BOC-harmol-*O*-ethyl tosylate precursor (prepared in-house) was radiolabeled using [^18^F]fluoride in acetonitrile containing Kryptofix/K_2_CO_3_. The *N*-BOC-[^18^F]FEH was purified by HPLC (60% CH_3_CN:40% 0.1% aqueous Et_3_N; 2.5 mL/min, retention time 18 min) and purity was confirmed by radio TLC. Deprotection of *N*-BOC-[^18^F]FEH was carried out using trifluoroacetic acid in CH_2_Cl_2_ to provide [^18^F]FEH in modest radiochemical yields. [^18^F]FEH was taken in 5 mL of 0.9% sterile saline and passed through a 0.22 micron Millipore filter into a sterile dose vial.

### 2.5. Autoradiography

Brain slices were placed in six separate incubation chambers (3 chambers for PD, 3 chambers for CN, for total binding, competition with 10 μM clorgyline and competition with 10 μM deprenyl) and were allowed to thaw from −80 °C to ambient temperature for 10 min. Subsequently they were pre-incubated in PBS (pH 7.4) buffer at 25 °C for 10 min. Fresh PBS buffer (pH 7.4) containing [^18^F]FAZIN3 (111 kBq/cc) was added to all the chambers and incubated for 60 min. After incubation, slides were washed twice (each wash lasting 3 min) and were rinsed in cold deionized water, air dried and exposed to a phosphor screen for 24 h. Using the Optiquant program (Packard Instruments Co., Boston, MA, USA), regions of interest were drawn in cortical layers I–III, IV–VI and white matter regions and digital light units/mm^2^ (DLU/mm^2^) were used to quantify the percentage binding of [^18^F]FAZIN3. Drug challenge studies in the presence of clorgyline or deprenyl were also analyzed in the same manner.

Similarly slides were incubated in the buffer at room temperature for 10 min and then in buffer with 111 kBq/cc [^18^F]FEH at 25 °C for 1 h. Nonspecific binding was measured in the presence of 10 µM clorgyline. After incubation, slides were washed twice (each wash lasting one minute) with ice-cold buffer. Slides were then quickly dipped in cold deionized water, air dried and exposed to a phosphor screen for 24 h. The amount of binding was evaluated in digital light units (DLU/mm^2^).

### 2.6. Image Analysis

Using QuPath, LB annotations were drawn on UIHC images. QuPath was then used to train a machine learning classifier for LB for each of the brain slices. Measurements of the area of UIHC were obtained for AC (gray matter) and CC (white matter) regions of interest (ROIs). These measurements corresponded to the presence of LB. The UIHC images of each brain slice provided percent anti-ubiquitin area (μm^2^) indicative of LB in the AC cortical layers I–III, IV–VI and CC regions. Similarly, ROIs were drawn on [^18^F]FAZIN3 autoradiographs using Optiquant. Measurements of image intensity in DLU/mm^2^ of AC cortical layers I–III, IV–VI and CC regions were obtained. These correspond to [^18^F]FAZIN3 binding to MAO-A. Significant differences between groups were confirmed by Student’s t-test (*p* < 0.05). Correlation of UIHC area (μm^2^) with [^18^F]FAZIN3 for MAO-A area (in mm^2^) was carried out.

### 2.7. Statistical Analysis

Statistical differences and correlations between groups (PD GM versus CN GM, PD Layers I–III versus PD layers IV–VI and versus WM) were determined using Microsoft Excel 16. Statistical power was determined with Student’s t test and *p* values of <0.05 were considered to indicate statistical significance. Non-linear parametric analysis using Spearman’s coefficient was used for correlation analysis between [^18^F]FAZIN3 autoradiography and UIHC LB staining measured in QuPath.

## 3. Results

### 3.1. Anti-Ubiquitin and α-Synuclein Immunohistochemistry

**Figure 1:**
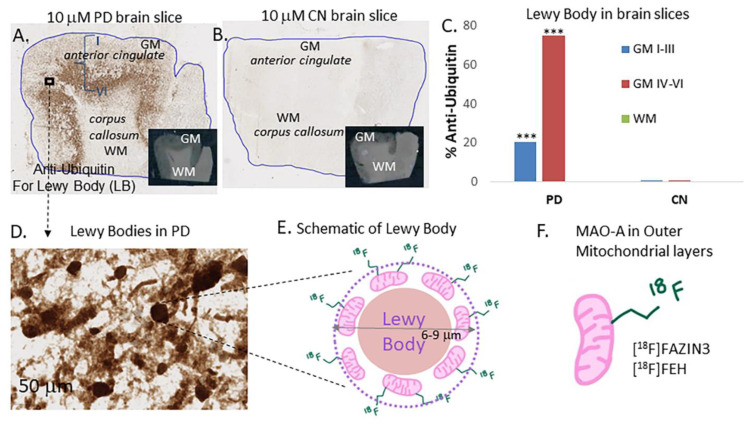
Lewy body: (**A**). Brain slice from PD subject #11-88 stained with anti-ubiquitin (UIHC) showing LB in the grey matter (GM) regions (inset shows scan of 10 μm brain slice); (**B**). Brain slice from control subject shows lack of staining by anti-ubiquitin, confirming absence of LB (inset shows scan of 10 μm brain slice); (**C**). Plot shows percent of anti-ubiquitin in GM cortical layers I–III and GM layers IV–VI in PD subjects and control subjects. High percentages of LB were present in PD IV–VI while lower amounts were present in PD I–III and no LB in PD WM. Control subjects GM and WM showed no LB (*** *p* < 0.001 for cortical layers versus WM and CN GM); (**D**). Magnified view of GM showing LB in the PD brain slice; (**E**). Schematic view of LB in the inset with a diameter range of 6 to 9 microns consistent with reported measures showing LB surrounded by mitochondria, and a potential target for fluorine-18 labeled PET imaging agent; (**F**). Monoamine oxidase A in the outer layer of mitochondria as a potential biomarker, using [^18^F]FAZIN3 and [^18^F]FEH.

All brain samples (6 PD and 6 CN) were immunostained with anti-ubiquitin (UIHC) for Lewy bodies. All PD brains were positively stained with UIHC (Figure 1A), while the CN brains did not contain any LB (Figure 1B). Inner cortical layers, IV–VI had greater percent of LB compared to outer cortical layers, I–III. Control brains and white matter (WM) regions had no UIHC staining, suggesting a lack of LB (Figure 1C). Closer examination of PD brain slice at 50 μm shows abundant LB (Figure 1D). All PD brain samples were confirmed for the presence of LB. Figure 1A shows the PD AC (inset shows brain slice with GM and WM) with outer cortical layers (I–III) having approximately 21% UIHC while inner layers (IV–VI) had >75% UIHC (Figure 1C). Lewy bodies were absent in the CN brain slice as expected and all CN subjects’ cortex (grey matter; GM) had <1% UIHC (Figure 1B). Presence of LB was ascertained by closer inspection of UIHC images (Figure 1D), with diameter ranges of 6–9 μm. A schematic of the LB surrounded by mitochondria which are known to contain MAO-A is shown in Figure 1E. This LB bound mitochondrial MAO-A as well as cytoplasmic mitochondrial MAO-A is targeted by [^18^F]FAZIN3 and [^18^F]FEH (Figure 1F).

### 3.2. [^18^F]FAZIN3 Post-Mortem Human PD Brain Autoradiography

**Figure 2:**
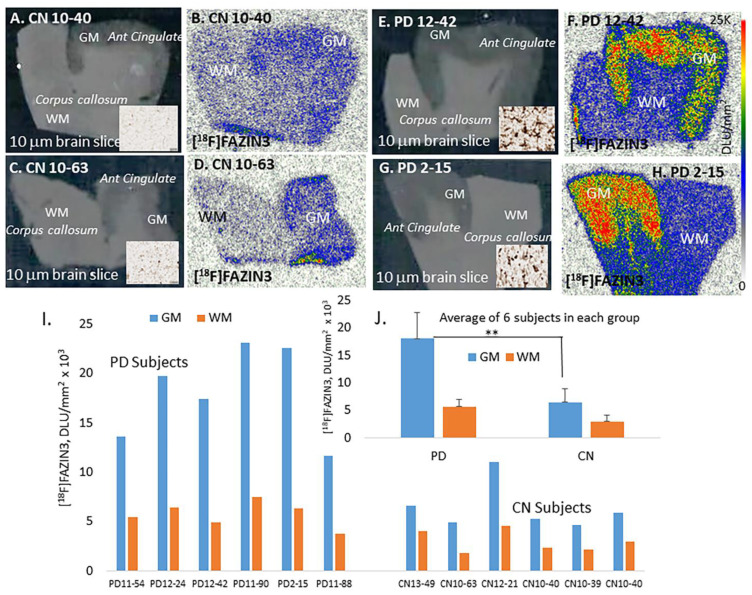
[^18^F]FAZIN3 binding to MAO-A: (**A**,**C**). Brain slices (10 μm) of CN subjects show absence of LB in the GM regions of anterior cingulate (inset images UIHC stained); (**B**,**D**). Binding of [^18^F]FAZIN3 in the CN brain GM and lower levels in corpus callosum, WM. (**E**,**G**). Brain slices (10 μm) of PD subjects showing abundant LB in the GM regions of anterior cingulate (inset images UIHC stained). (**F**,**H**). Binding of [^18^F]FAZIN3 in the PD brain GM was significantly greater compared to that of CN GM. [^18^F]FAZIN3 binding was seen in all CN and PD subject, with GM showing higher levels compared to WM. PD brains showed greater [^18^F]FAZIN3 in GM regions compared to control subjects. (**I**). Plot of GM and WM of all subjects show PD subjects exhibited significantly higher binding of [^18^F]FAZIN3 in GM compared to CN. (**J**). Plot shows averages of all PD and CN subjects (** *p* < 0.01 for PD GM versus CN WM). Ratio of PD GM/WM = 3.15 whereas CN GM/WM = 2.17. Gray matter of PD subjects had >100% increase in [^18^F]FAZIN3 compared to control subjects.

Autoradiographic studies with [^18^F]FAZIN3 in CN and PD subjects are shown in Figure 2. The GM and WM were clearly delineated in the autoradiographic images of [^18^F]FAZIN3 in all subjects. [^18^F]FAZIN3 exhibited binding across the cortical layers in all subjects. Figure 2A,C show two CN subjects brain slices (insets show absence of LB) and their corresponding [^18^F]FAZIN3 binding in Figure 2B,D. Figure 2E,G show two PD subjects brain slices (insets show presence of LB) and their corresponding [^18^F]FAZIN3 binding in Figure 2F,H. A distinct difference can be seen in the levels of [^18^F]FAZIN3 binding between the CN subjects (Figure 2B,D) compared to the PD subjects (Figure 2F,H). All CN subjects exhibited lower [^18^F]FAZIN3 binding in the GM compared to the PD subjects (Figure 2I). Using the Optiquant program (Packard Instruments Co., Boston, MA, USA), regions of interest were drawn and digital light units/mm^2^ (DLU/mm^2^) were used to quantify [^18^F]FAZIN3 binding. Figure 2J shows average [^18^F]FAZIN3 binding of all 6 PD and CN subjects in GM and WM regions. Ratio of PD GM/CN GM = 2.78, indicating >200% increase in PD, while average GM/WM = 2.18 for all CN subjects and average GM/WM = 3.15 for all PD subjects, suggesting a 45% increase in PD using ratios, which is more relevant to in vivo imaging where typically a reference region for quantification. All PD subjects showed lack of [^18^F]flotaza binding [30] confirming absence of Aβ plaques, and absence of [^125^I]IPPI binding [31], confirming absence of neurofibrillary tangles (NFT). Thus, an increase in MAO-A in the anterior cingulate of PD brains compared to CN brains was observed.

In order to further ascertain this increase in MAO-A in the six PD subjects, we prepared [^18^F]FEH, a known fluorine-18 analog of [^11^C]Harmine MAO-A radiotracer [20], and tested the CN and PD subjects’ brains. An increase in the binding of [^18^F]FEH in PD brains was observed (Figure S1).

### 3.3. [^18^F]FAZIN3 Drug Effects Post-Mortem Human PD Brain

**Figure 3:**
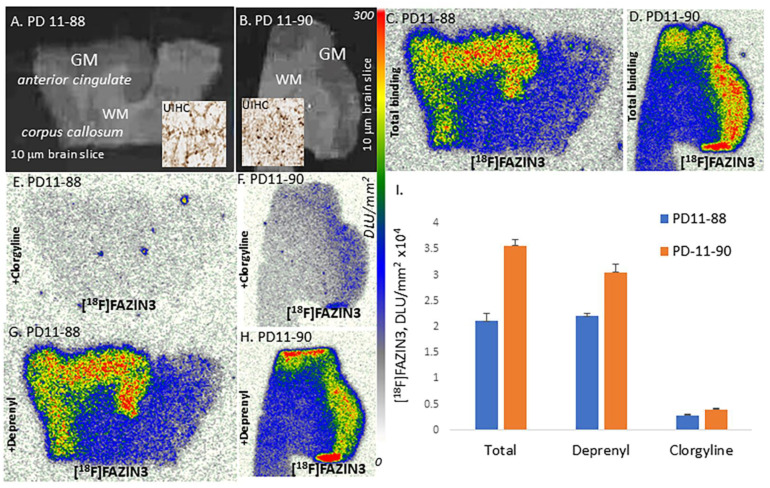
MAO drug effects on [^18^F]FAZIN3 and LB correlation: (**A**). PD#11-88, 10 μm brain slice showing anterior cingulate (GM) and corpus callosum (CC); inset shows UIHC stained GM. (**C**). MAO-A in GM labeled by [^18^F]FAZIN3 in adjacent section of PD#11-88. (**E**). MAO-A drug, clorgyline 10 μM displaced [^18^F]FAZIN3 in adjacent section of PD#11-88. (**G**). MAO-B drug, deprenyl 10 μM had little effect on [^18^F]FAZIN3. (**B**,**D**,**F**,**H**). Similar set of results with second subject PD#11-90 of [^18^F]FAZIN3 total binding, clorgyline displacement and no effect by deprenyl. (**I**). Plot of [^18^F]FAZIN3 total binding, in the presence of deprenyl and clorgyline in PD subjects.

Binding of [^18^F]FAZIN3 to MAO-A was ascertained by competition with clorgyline, a known MAO-A irreversible inhibitor, and (*R*)-deprenyl, a known irreversible MAO-B inhibitor. Shown in Figure 3 are two PD subjects brain sections (Figure 3A,B) with UIHC insets showing presence of LB. Total binding of [^18^F]FAZIN3 in these PD subjects is shown in Figure 3C,D. Distinct binding of [^18^F]FAZIN3 in the cortical regions is evident. Clorgyline at 1 μM concentration displaced over 80–90% of [^18^F]FAZIN3 binding (Figure 3E,F), suggesting binding of [^18^F]FAZIN3 to MAO-A. In the presence of 1 μM (*R*)-deprenyl, there was little effect on the binding of [^18^F]FAZIN3, as seen in Figure 3G,H. Similar effects of both clorgyline and (*R*)-deprenyl were observed in the CN brains, confirming that [^18^F]FAZIN3 binds to MAO-A in PD (Figure 3I).

**Figure 4:**
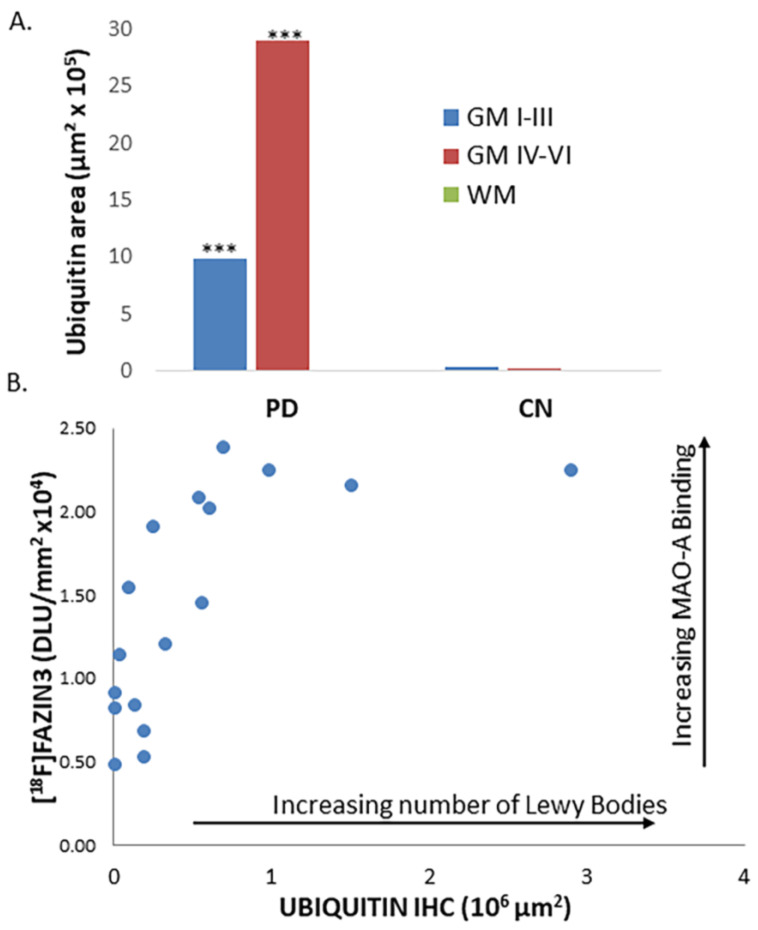
(**A**). Quantitation of LB in anti-ubiquitin stained brain sections of PD and CN subjects (*** *p* < 0.001 for cortical layers versus WM and CN GM). (**B**). Correlation plot of anti-ubiquitin and [^18^F]FAZIN3 in all human subjects (Spearman’s correlation coefficient, ρ = 0.83 and *p* < 0.001). Plot shows an increase in [^18^F]FAZIN3 binding to MAO-A present in the mitochondria surrounding the LB, with a plateauing effect at UIHC 10^6^ μm^2^.

Using QuPath, the UIHC stained brain sections were analyzed to provide UIHC μm^2^ (or LB μm^2^) in CN and PD brain regions of WM and GM (Figure 4A). Correlation of the UIHC μm^2^ with [^18^F]FAZIN3 binding to MAO-A in DLU/mm^2^ is shown in Figure 4B. There is an increase in [^18^F]FAZIN3 binding as the UIHC μm^2^ increases with a plateauing effect of approximately 10^6^ mm^2^ UIHC (Spearman’s correlation coefficient, ρ = 0.83 and *p* < 0.001). These mitochondria are labeled by [^18^F]FAZIN3 and because of their localized higher concentration in PD (compared to CN subjects), higher MAO-A binding by [^18^F]FAZIN3 is observed.

## 4. Discussion

Lewy bodies (LB) are present in the cell bodies in various brain regions in PD patients [5,32]. A greater understanding of the formation of LB is emerging in which there is retrograde axonal transport of α-synuclein fibrils and neurites to the cell body which may then trigger an aggresome response, resulting in the formation of LB [20,33]. Many proteins, α-synuclein fibrils and aggregates, and macrophages are known to be present in LB and are encircled by mitochondria (Figure 1). Monoamine oxidase-A, present in the outer layers of the mitorchondria, may play a significant role in the pathogenesis of neurodegeneration causing neurotransmitter breakdown, increased production of reactive oxygen species and possible roles in microglia activation and inflammation [8,34].

Reversible MAO-A PET radiotracer [^11^C]harmine was used to study depression [19]. In a PET study comparing healthy controls with depressed individuals, a 34% increase in distribution volume of [^11^C]harmine was measured in several brain regions, including anterior cingulate. This was considered indicative of an increase in MAO-A (either more MAO-A per mitochondrion or more mitochondria) causing a decrease in monoaminergic neurotransmitters, such as dopamine, serotonin and norepinephrine. A molecular mechanism causing an increase in MAO-A in affective disorders is not clearly understood. PET imaging studies of MAO-A in PD have not been carried out.

The harmine-related fluorine-18 analog [^18^F]FEH has only been used in small animal imaging. Rapid metabolism and poor brain penetration may have limited further development of [^18^F]FEH analogs. The lack of reversible fluorine-18 PET imaging radiotracers for MAO-A has been an impediment and there is now ongoing effort to develop such MAO-A PET imaging agents [18]. We have developed the fluoroalkyl azaindole, [^18^F]FAZIN3 as a new PET radiotracer that binds reversibly and selectively to MAO-A.

In anterior cingulate, LB may be used to predict cognitive deficits in PD [25] and also has been shown to contain a high amount of MAO-A [21]. Therefore, our goal in this initial study was to evaluate [^18^F]FAZIN3 LB in the anterior cingulate of well characterized PD subjects and normal controls (Table 1). The anterior cingulate of all PD subjects were positively stained using anti-ubiquitin IHC compared to controls (Figure 1). Using QuPath, percent of LB was quantified and was outer cortical layers (I–III) of PD had 21% UIHC while inner layers (IV–VI) had >75% UIHC, as reported previously for LB distribution in cortical layers [25]. Normal control anterior cingulate and corpus callosum had essentially no UIHC, suggesting a lack of LB.

Binding of [^18^F]FAZIN3 was seen in the anterior cingulate of all subjects which was greater than the white matter, corpus callosum (Figure 2). However, the extent of [^18^F]FAZIN3 binding in the PD brains (Figure 2F,H) was significantly greater compared to the normal controls (Figure 2B,D). The ratio of [^18^F]FAZIN3 in PD for AC/CC was 3.57, while in CN subjects it was AC/CC = 2.24, suggesting a 59% increase if the ratios were used. If only the anterior cingulate was compared between the PD and CN anterior cingulate, there was >100% increase in the binding of [^18^F]FAZIN3 in PD subjects compared to controls. This suggests a significantly higher MAO-A in the anterior cingulate of PD subjects. This increase is very significant and is greater than reported previously in depressed patients using [^11^C]harmine [19]. This is indicative of an increase in MAO-A in PD (either more MAO-A per mitochondrion or more mitochondria or more dysphoric mitochondria in PD). It should be noted that the PD subjects did not report depression as a comorbidity (Table 1).

Since both MAO-A and MAO-B have been key enzymes in the degradation of neurotransmitters in the brain, selectivity of [^18^F]FAZIN3 was ascertained by using competition with clorgyline, a MAO-A irreversible inhibitor and (*R*)-deprenyl, a MAO-B irreversible inhibitor [15,16]. Shown in Figure 3 are two PD subjects showing selectivity of [^18^F]FAZIN3 for MAO-A (by virtue of being displaced >90% by clorgyline). Because of a lack of effect of (*R*)-deprenyl on [^18^F]FAZIN3, it may be safely inferred that the increased binding of [^18^F]FAZIN3 in the PD subjects are elevations in MAO-A binding. The findings of [^18^F]FAZIN3 in the PD patient shown in Figure 1H were further supported by the experiments of the known MAO-A radiotracer, [^18^F]FEH which demonstrated binding in the anterior cingulate of PD brain of the same patient (Figure S1C) and was displaced by clorgyline (Figure S1D).

Correlation of UIHC with [^18^F]FAZIN3 across all subjects in the gray matter and white matter regions (Figure 4B) suggests a relationship of an increase in MAO-A with UIHC, with a plateau around 10^6^ μm^2^. Since [^18^F]FAZIN3 was used at tracer levels, it is unlikely there is a saturation effect of the available MAO-A sites. A correlation of LB per mm^2^ with MAO-A radiotracer binding may potentially be used to assess LB load measurement and thus staging of the disease [25,35]. A molecular mechanism causing an increase in MAO-A in PD may be related to a potential mitochondrial dysfuntion caused by the formation of LB. There are ongoing efforts to understand the role of α-synuclein fibrils and neurites in the formation of LB in PD [6,33]. Figure S2 shows our preliminary studies of anti-α-synuclein IHC of PD brain slices (Figure S2B) confirming the presence of Lewy neurites (Figure S2C) and presence of α-synuclein in LB (Figure S2D). The distribution of anti-α-synuclein IHC across the cortical layers was sparse and was not as discrete between the cortical layers as was the case with UIHC (Figure 1A versus Figure S2B). Although α-synuclein aggregates have been suggested to be involved in LB formation [36,37], more careful assessment of α-synuclein aggregate distribution in the anterior cingulate will have to be carried out in order to establish such links in the UIHC. However, α-synuclein may have a role in upregulating MAO-A; as suggested [8], the distribution of MAO-A appears to correlate more strongly with UIHC.

Studies have suggested that LB are formed initially in the SN and progressively spread to cortical regions. Although LB are present in PD subjects, their causative effect on PD is debatable, since increasing amounts of LB in SN has not necessarily shown neuronal loss [32,38]. Our approach to measure MAO-A may yield additional cellular information on cellular oxidative stress leading up to the formation of LB. Nevertheless, the presence of abundant mitochondria (normal and dysphoric) in LB provides a good surrogate biomarker for LB and thus for PD. Imaging probes have been used for PD that measure loss of metabolism and other protein targets along the neurotransmitter-receptor pathways and are thus “cold spot” imaging agents [39,40,41,42]. Increased [^18^F]FAZIN3 binding in PD with the increased presence of LB is a novel “hot spot” imaging approach (Figure 1 and Figure 2).

Limitations of the study include small number of subjects in advanced stages of PD. A larger study with more subjects at different disease stages is needed in order to ascertain the correlation of MAO-A imaging with UIHC. Other brain regions which are known to contain LB in PD, such as substantia nigra, need to be studied to assess MAO-A increases. Although LB play a significant role in PD, the role of MAO-A in neurodegeneration associated with PD needs further studies. Finally, an in vivo PET imaging study for MAO-A measures in PD will have to be done, since results reported here are only in post-mortem brains.

## 5. Conclusions

Our results suggest that MAO-A imaging is a potential surrogate biomarker for PD and LB. Several PET imaging probes have been used for PD that measure loss of monoaminergic targets and are thus “cold spot” imaging agents [43]. Increased [^18^F]FAZIN3 binding in PD with the presence of LB is a novel “hot spot” imaging approach. Our results suggests that increased levels of MAO-A in LB due to increased mitochondria in LB may be a sensitive prodromal tool in earlier diagnosis of PD. The value of MAO-A imaging in LB may be extended to other neurodegenerative conditions such as LB dementia (LBD).

## Figures and Tables

**Table 1:** Patient samples and data *.

ID	Pathology	Gender	Age Expired	Brain Region ^1^	Braak Score	LB Stage	LB Density ^2^
10-39	CN	Male	93	Ant Cingulate	I	0	No LB
10-63	CN	Male	79	Ant Cingulate	II	0	No LB
10-70	CN	Male	74	Ant Cingulate	I	0	No LB
12-21	CN	Female	88	Ant Cingulate	II	0	No LB
13-40	CN	Male	73	Ant Cingulate	II	0	No LB
13-49	CN	Female	75	Ant Cingulate	II	0	No LB
02-15	PD	Female	78	Ant Cingulate	III	IV	31
11-54	PD	Female	76	Ant Cingulate	III	III	28
11-88	PD	Male	89	Ant Cingulate	III	III	25
11-90	PD	Male	78	Ant Cingulate	II	III	28
12-29	PD	Male	86	Ant Cingulate	III	IV	32
12-42	PD	Male	69	Ant Cingulate	II	IV	29

* Frozen brain samples were obtained from Banner Sun Health Research Institute (BHRI), Sun City Arizona [28]; CN = cognitively normal and may include mild cognitive impairment (MCI) subjects; PD = Parkinson’s disease. LB = Lewy body; ^1^ Brain tissue contained anterior cingulate and corpus callosum; ^2^ Sum of LB from different brain regions of the subjects obtained from BHRI.

## Data Availability

The data that support the findings of this study are available for discussions from the corresponding author upon reasonable request.

## Supplementary Materials

The following supporting information can be downloaded at: https://www.mdpi.com/article/10.3390/cells11244000/s1, Figure S1: [^18^F]FEH binding in PD human subjects: (**A**). PD2-15 10 μm brain slice showing anterior cingulate (GM) and corpus callosum (CC); (**B**). MAO-A in GM labeled by [^18^F]FEH in adjacent section of PD2-15. (**C**). MAO-A drug, clorgyline 10 μM displaced [^18^F]FEH in adjacent section of PD2-15; inset shows UIHC stained GM; Figure S2: Anti-α-Synuclein and [^18^F]FAZIN3: (**A**). Binding of [^18^F]FAZIN3 in PD 12-42 brain slice. (**B**). Adjacent PD 12-42 brain slice labeled with anti-α-synuclein showing labeling of GM and suggesting presence of aggregated α-synuclein. (**C**). Closer view (50 μm) shows presence of Lewy neurites. (**D**). Presence of anti-α-synuclein LB at 20 μm, similar to LB observed with anti-ubiquitin.
