# Supplementary material for: Elevated Monoamine Oxidase-A in Anterior Cingulate of Post-Mortem Human Parkinson’s Disease: A Potential Surrogate Biomarker for Lewy Bodies?
Source: Cells. 2022 Dec 10;11(24):4000. doi: 10.3390/cells11244000 (PMC9777299; doi:10.3390/cells11244000)
Supplement: Supplementary file 1 [file cells-11-04000-s001.zip › cells-2080743-supplementary.pdf]

CELLS

Article

Elevated Monoamine Oxidase-A in Anterior Cingulate of Post-Mortem Human Parkinson's Disease: A Potential Surrogate Biomarker for Lewy Bodies?

Jogeshwar Mukherjee [1*], Reisha M. Ladwa [1], Christopher Liang [1], and Amina U. Syed [1]

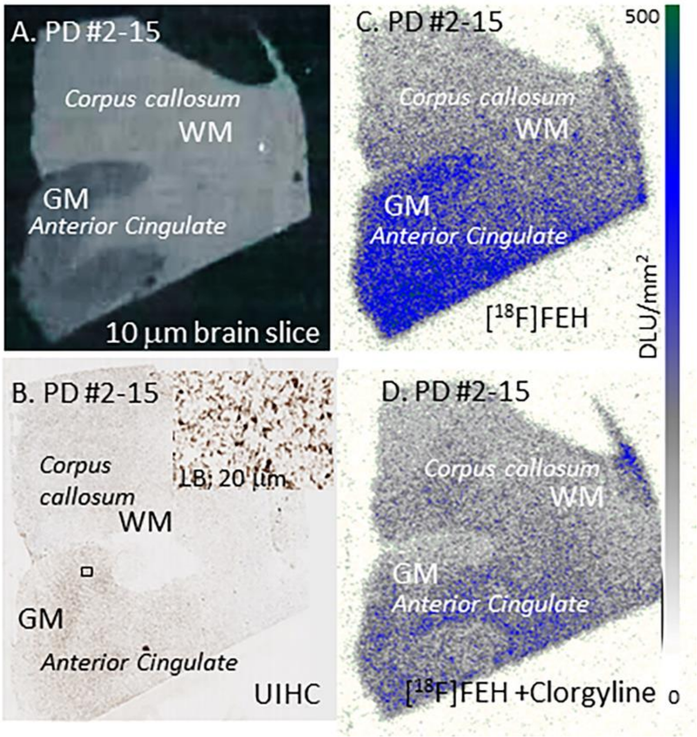

Figure-S1: [^{18}F]FEH binding in PD human subjects: (A). PD#2-15 10 μm brain slice showing anterior cingulate (GM) and corpus callosum (CC); (B). Adjacent PD#2-15 10 μm brain slice UIHC stained, inset shows presence of LB in GM. (C). MAO-A in GM labeled by [^{18}F]FEH in adjacent section of PD#2-15. (D). MAO-A drug, clorgyline 10 μM displaced [^{18}F]FEH in adjacent section of PD#2-15.

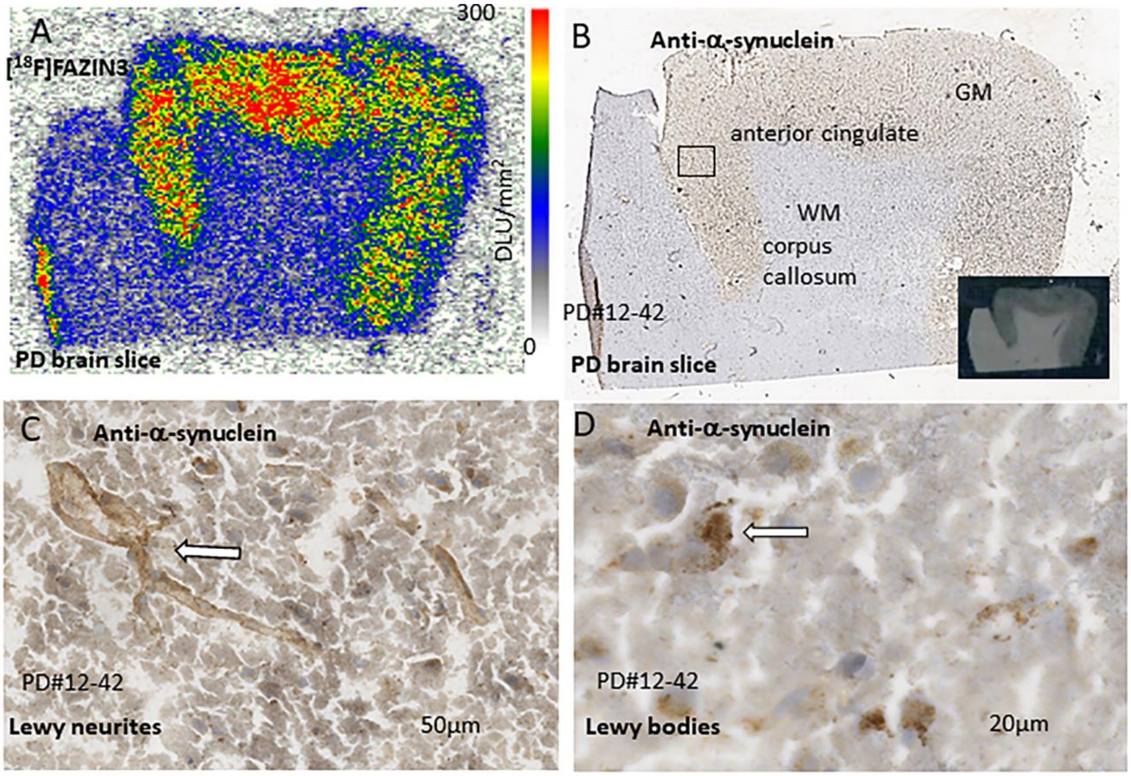

Figure-S2: Anti-α-Synuclein and [^{18}F]FAZIN3: (A) Binding of [^{18}F]FAZIN3 in PD #12-42 brain slice. (B). Adjacent PD #12-42 brain slice labeled with anti-α-synuclein showing labeling of GM and suggesting presence of aggregated α-synuclein. (C). Closer view (50 µm) shows presence of Lewy neurites. (D). Presence of anti-α-synuclein LB at 20 µm, similar to LB observed with anti-ubiquitin.
